# A bag-of-words approach for *Drosophila *gene expression pattern annotation

**DOI:** 10.1186/1471-2105-10-119

**Published:** 2009-04-21

**Authors:** Shuiwang Ji, Ying-Xin Li, Zhi-Hua Zhou, Sudhir Kumar, Jieping Ye

**Affiliations:** 1 Center for Evolutionary Functional Genomics, The Biodesign Institute, Arizona State University, Tempe, AZ 85287, USA; 2 Department of Computer Science and Engineering, Arizona State University, Tempe, AZ 85287, USA; 3 National Key Laboratory for Novel Software Technology, Nanjing University, Nanjing 210093, PR China; 4 School of Life Sciences, Arizona State University, Tempe, AZ 85287, USA

## Abstract

**Background:**

*Drosophila *gene expression pattern images document the spatiotemporal dynamics of gene expression during embryogenesis. A comparative analysis of these images could provide a fundamentally important way for studying the regulatory networks governing development. To facilitate pattern comparison and searching, groups of images in the Berkeley *Drosophila *Genome Project (BDGP) high-throughput study were annotated with a variable number of anatomical terms manually using a controlled vocabulary. Considering that the number of available images is rapidly increasing, it is imperative to design computational methods to automate this task.

**Results:**

We present a computational method to annotate gene expression pattern images automatically. The proposed method uses the bag-of-words scheme to utilize the existing information on pattern annotation and annotates images using a model that exploits correlations among terms. The proposed method can annotate images individually or in groups (e.g., according to the developmental stage). In addition, the proposed method can integrate information from different two-dimensional views of embryos. Results on embryonic patterns from BDGP data demonstrate that our method significantly outperforms other methods.

**Conclusion:**

The proposed bag-of-words scheme is effective in representing a set of annotations assigned to a group of images, and the model employed to annotate images successfully captures the correlations among different controlled vocabulary terms. The integration of existing annotation information from multiple embryonic views improves annotation performance.

## Background

Study of the interactions and functions of genes is crucial to deciphering the mechanisms governing cell-fate differentiation and embryonic development. The DNA microarray technique is commonly used to measure the expression levels of a large number of genes simultaneously. However, this technique primarily documents the average expression levels of genes, with information on spatial patterns often unavailable [1,2]. In contrast, The RNA *in situ *hybridization uses gene-specific probes and illuminates the spatial patterns of gene expression precisely. Recent advances in this high-throughput technique have generated spatiotemporal information for thousands of genes in organisms such as *Drosophila *[1,3] and mouse [4]. Comparative analysis of the spatiotemporal patterns of gene expression can potentially provide novel insights into the functions and interactions of genes [5-7].

**Figure 1:**
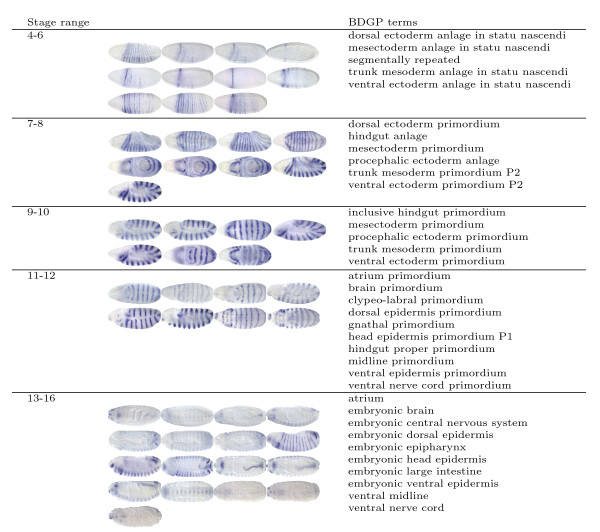
**Sample image groups and their associated terms in the BDGP database  for the segmentation gene *engrailed *in 5 stage ranges**.

The embryonic patterning of *Drosophila melanogaster *along the anterior-posterior and dorsal-ventral axes represents one of the best understood examples of a complex cascade of transcriptional regulation during development. Systematic understanding of the mechanisms underlying the patterning is facilitated by the comprehensive atlas of spatial patterns of gene expression during *Drosophila *embryogenesis, which has been produced by the *in situ *hybridization technique and documented in the form of digital images [1,8]. To provide flexible tools for pattern searching, the images in the Berkeley *Drosophila *Genome Project (BDGP) high-throughput study are annotated with anatomical and developmental ontology terms using a controlled vocabulary (CV) [1] (Figure 1). These terms integrate the spatial and temporal dimensions of gene expression by describing a developmental "path" that documents the dynamic process of *Drosophila *embryogenesis [1,2]. Currently, the annotation is performed manually by human curators. However, the number of available images is now rapidly increasing [5,9-11]. It is therefore tempting to design computational methods to automate this task.

**Figure 2:**
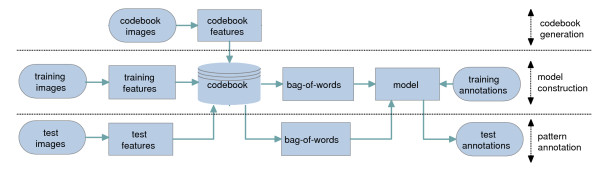
**Flowchart of the proposed method for annotating gene expression patterns**.

The particular nature of this problem determines that some challenging questions need to be addressed while designing the automated method. Owing to the effects of stochastic processes during development, no two embryos develop identically. Also, the quality of the obtained data is limited by current image processing techniques. Hence, the shape and position of the same embryonic structure may vary from image to image. Indeed, this has been considered as one of the major impediments to automate this task [1]. Thus, invariance to local distortions in the images is an essential requirement for the automatic annotation system. Furthermore, gene expression pattern images are annotated collectively in small groups using a variable number of terms in the original BDGP study. Images in the same group may share certain anatomical and developmental structures, but all terms assigned to a group of images do not apply to every image in the group. This requires the development of approaches that can retain the original group membership information of images, because we need to test the accuracy of the new method using existing (and independent) annotation data. Prior work on this task [12] ignored such groups and assumed that all terms are associated with all images in a group, which may adversely impact their effectiveness for use on the BDGP data. Finally, the *Drosophila *embryos are 3D objects, and they are documented as 2D images taken from multiple views. Since certain embryonic structures can only be seen in specific two-dimensional projections (views), it is beneficial to integrate images with different views to make the final annotation. In this article we present a computational method for annotating gene expression pattern images. This method is based on the bag-of-words approach in which invariant visual features are first extracted from local patches on the images, and they are then quantized to form the bag-of-words representation of the original images. This approach is known to be robust to distortions in the images [13,14], and it has demonstrated impressive performance on object recognition problems in computer vision [15] and on image classification problems in cell biology [16]. In our approach, invariant features are first extracted from local patches on each image in a group. These features are then quantized based on precomputed "visual codebooks", and images in the same group with the same view is represented as a bag-of-words. Thus, our approach can take advantage of the group membership information of images as in the BDGP study. To integrate images with different views, we propose to construct a separate codebook for images with each view. Then image groups containing images with multiple views can be represented as multiple bags, each containing words from the corresponding view. We show that multiple bags can be combined to annotate the image group collectively. After representing each image group as multiple bags of words, we employ a classification model [17] developed recently to annotate the image groups. This model [17] can exploit the correlations among different terms, leading to improved performance. Experimental results on the gene expression pattern images obtained from the FlyExpress database  show that the proposed approach outperforms other methods consistently. Results also show that integration of images with multiple views improves annotation performance. The overall flowchart of the proposed method is depicted in Figure 2.

## Methods

**Figure 3:**
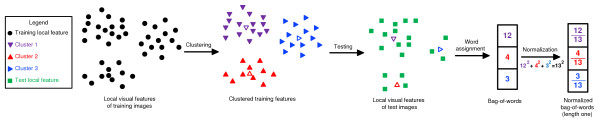
**Illustration of the bag-of-words approach**. First, a visual codebook is constructed by applying a clustering algorithm to a subset of the local features from training images, and the center of each cluster is considered as a unique "visual words" in the codebook. Each local feature in a test image is then mapped to the closest visual word, and each test image is represented as a (normalized) histogram of visual words.

The proposed method is based on the bag-of-words approach, which was originally used in text mining, and is now commonly employed in image and video analysis problems in computer vision [15,18-20]. In this approach, invariant features [21] are first extracted from local regions on images or videos, and a visual codebook is constructed by applying a clustering algorithm on a subset of the features where the cluster centers are considered as "visual words" in the codebook. Each feature in an image is then quantized to the closest word in the codebook, and an entire image is represented as a global histogram counting the number of occurrences of each word in the codebook. The size of the resulting histogram is equal to the number of words in the codebook and hence the number of clusters obtained from the clustering algorithm. The codebook is usually constructed by applying the flat *k*-means clustering algorithm or other hierarchical algorithms [14]. This approach is derived from the bag-of-words models in text document categorization, and is shown to be robust to distortions in images. One potential drawback of this approach is that the spatial information conveyed in the original images is not represented explicitly. This, however, can be partially compensated by sampling dense and redundant features from the images. The bag-of-words representation for images is shown to yield competitive performance on object recognition and retrieval problems after some postprocessing procedures such as normalization or thresholding [14,15]. The basic idea behind the bag-of-words approach is illustrated in Figure 3.

For our problem, the images are annotated collectively in small groups in the BDGP database. Hence, we propose to extract invariant visual features from each image in a group and represent the images in the same group with the same view as a bag of visual words. The 3D nature of the embryos and the 2D layout of the images determine that certain body parts can only be captured by images taken from certain views. For example, the body part "ventral midline" can only be identified from images taken from the ventral view. Hence, one of the challenges in automated gene expression pattern annotation is the integration of images with different views. We propose to construct a separate codebook for images with each view and quantize the image groups containing images with multiple views as multiple bags of visual words, one for each view. The bags for multiple views can then be concatenated to annotate the image groups collectively. After representing each image group as a bag-of-words, we propose to apply a multi-label classification method developed recently [17] that can extract shared information among different terms, leading to improved annotation performance.

### Feature extraction

**Figure 4:**
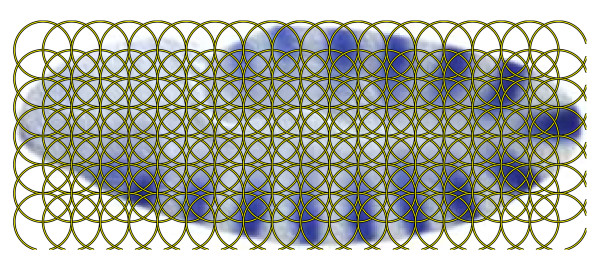
**Illustration of the image patches on which the SIFT features are extracted**. We extract local features on regular patches on the images where the radius and spacing of the regular patches are set to 16 pixels.

The images in the FlyExpress database have been standardized semi-automatically, including alignment. Three common methods for generating local patches on images are those based on affine region detectors [22], random sampling [23], and regular patches [24]. We extract dense features on regular patches on the images, since such features are commonly used for aligned images. The radius and spacing of the regular patches are set to 16 pixels in the experiments (Figure 4). Owing to the limitations of image processing techniques, local variations may exist on the images. Thus, we extract invariant features from each regular patch. In this article, we apply the SIFT descriptor [21,25] to extract local visual features, since it has been applied successfully to other image-related applications [21]. In particular, each feature vector is computed as a set of orientation histograms on 4 × 4 pixel neighborhoods, and each histogram contains 8 bins. This leads to a SIFT feature vector with 128 (4 × 4 × 8) dimensions on each patch. Note that although the invariance to scale and orientation no longer exists since we do not apply the SIFT interest point detector, the SIFT descriptor is still robust against the variance of position, illumination, and viewpoint [25].

### Codebook construction

In this article, we consider images taken from the lateral, dorsal, and ventral views, since the number of images from other intermediate views is small. For each stage range, we build a separate codebook for images with each view. Since the visual words of the codebooks are expected to be used as representatives of the embryonic structures, the images used to build the codebooks should contain all the embryonic structures that the system is expected to annotate. Hence, we extract codebook images in a way so that each embryonic structure appears in at least a certain number of images. This number is set to 10, 5, and 3 for codebooks of lateral, dorsal, and ventral images, respectively, based on the total number of images with each view (Table 1). The SIFT features computed from regular patches on the codebook images are then clustered using the *k*-means algorithm. Since this algorithm depends on the initial centers, we repeat the algorithm with ten random initializations from which the one resulting in the smallest summed within-cluster distance is selected. We study the effect of the number of clusters (i.e., the size of the codebook) on the performance below and set this number to 2000, 1000, and 500 for lateral, dorsal, and ventral images, respectively.

**Table 1:** Summary of the statistics of the BDGP images.

Stage range	1–3	4–6	7–8	9–10	11–12	13–16
Size of CV	2	42	24	45	102	119
# of image groups	1783	1081	877	1072	2113	2816
# of lateral images	2395	4173	1954	2153	7441	7564
# of dorsal images	127	847	812	1174	2837	6480
# of ventral images	3	559	319	213	1032	1390

An image group is a group of gene expression pattern images of a particular gene at a particular stage range.

### Pattern representation

After the codebooks for all views are constructed, the images in each group are quantized separately for each view. In particular, features computed on regular patches on images with a certain view are compared with the visual words in the corresponding codebook, and the word closest to the feature in terms of Euclidean distance is used to represent it. Then the entire image group is represented as multiple bags of words, one for each view. Since the order of the words in the bag is irrelevant as long as it is fixed, the bag can be represented as a vector counting the number of occurrences of each word in the image group. Let *c*_1_,⋯,*c*_*m *_∈ ℝ^*d *^be the *m *cluster centers (codebook words) and let *v*_1_,⋯,*v*_*n *_∈ ℝ^*d *^be the *n *features extracted from images in a group with the same view where *d *is the dimensionality of the local features (*d *= 128 for SIFT). Then the bag-of-words vector *w *is *m*-dimensional, and the *k*-th component *w*_*k *_of *w *is computed as



where *δ*(*a, b*) = 1 if *a *= *b*, and 0 otherwise, and ∥·∥ denotes the vector 2-norm. Note that , since each feature is assigned to exactly one word.

Based on this design, the vector representation for each view can be concatenated so that the images in a group with different views are integrated (Figure 3). Let *w*^*l*^, *w*^*d*^, and *w*^*v *^be the bag-of-words vector for images in a group with lateral, dorsal, and ventral views, respectively. Then the bag-of-words vector *w *for the entire image group can be represented as



To account for the variability in the number of images in each group, we normalize the bag-of-words vector to unit length. Note that since not all the image groups contain images from all views, the corresponding bag-of-words representation is a vector of zero if a specific view is absent.

### Pattern annotation

After representing each image group as a global histogram using the bag-of-words representation, the gene expression pattern image annotation problem is reduced to a multi-label classification problem, since each group of images can be annotated with multiple terms. (We use the terminology "label" and "term" interchangably, since the former is commonly used in machine learning literature, and the latter is more relevant for our application). The multi-label problems have been studied extensively in the machine learning community, and one simple and popular approach for this problem is to construct a binary classifier for each label, resulting in a set of independent binary classification problems. However, this approach fails to capture the correlation information among different labels, which is critical for many applications such as the gene expression pattern image annotation problem where the semantics conveyed by different labels are correlated. To this end, various methods have been developed to exploit the correlation information among different labels so that the performance can be improved [17,26-29]. In [17], a shared-subspace learning framework has been proposed to exploit the correlation information in multi-label problems. We apply this formulation to the gene expression pattern image annotation problem in this article.

We are given a set of *n *input data vectors  ∈ ℝ^*d *^(*d *= 3500 if all of the three views are used) which are the bag-of-words representations of *n *image groups. Let the terms associated with each of the *n *image groups be encoded into the label indicator matrix *Y *∈ ℝ^*n *× *m *^where *m *is the total number of terms, and *Y*_*i*ℓ _= 1 if the *i*th image group has the ℓth term and *Y*_*i*ℓ _= -1 otherwise. In the shared-subspace learning framework proposed in [17], a binary classifier is constructed for each label to discriminate this label from the rest of them. However, unlike the approaches that build the binary classifiers independently, a low-dimensional subspace is assumed to be shared among multiple labels. The predictive functions in this framework consist of two parts: one part is contributed from the original data space, and the other part is derived from the shared subspace as follows:

(1)

where **w**_ℓ _∈ ℝ^*d *^and **v**_ℓ _∈ ℝ^*r *^are the weight vectors, Θ ∈ ℝ^*r *× *d *^is the linear transformation used to parameterize the shared low-dimensional subspace, and *r *is the dimensionality of the shared subspace. The transformation Θ is common for all labels, and it has orthonormal rows, that is ΘΘ^*T *^= *I*. In this formulation, the input data are projected onto a low-dimensional subspace by Θ, and this low-dimensional projection is combined with the original representation to produce the final prediction.

In [17] the parameters  and Θ are estimated by minimizing the following regularized empirical risk:

(2)

subject to the constraint that ΘΘ^*T *^= *I*, where *L *is some loss function,  = *Y*_*i*ℓ_, and *α *> 0 and *β *> 0 are the regularization parameters. It can be shown that when the least squares loss is used, the optimization problem in Eq. (2) can be expressed as

(3)

where *X *= [*x*_1_,⋯,*x*_*n*_]^*T *^∈ ℝ^*n *× *d *^is the data matrix, ∥·∥_*F *_denotes the Frobenius norm of a matrix [30], **u**_ℓ _= **w**_ℓ _+ Θ^*T*^**v**_ℓ_, *U *= [**u**_1_,⋯,**u**_*m*_], and *V *= [**v**_1_,⋯,**v**_*m*_]. The optimal Θ* can be obtained by solving a generalized eigenvalue problem, as summarized in the following theorem:

**Theorem 1 ***Let X, Y, and *Θ *be defined as above. Then the optimal *Θ *that solves the optimization problem in Eq. (3) can be obtained by solving the following trace maximization problem:*

(4)

*where S*_1 _*and S*_2 _*are defined as:*

(5)

(6)

(7)

For high-dimensional problems where *d *is large, an efficient algorithm for computing the optimal Θ is also proposed in [17]. After the optimal Θ is obtained, the optimal values for  can be computed in a closed form.

## Results and discussion

We report and analyze the experimental results on gene expression pattern annotation in this section. We also demonstrate the performance improvements achieved by integrating images with multiple views and study the effect of the codebook size on the annotation performance. The performance for each individual term is also presented and analyzed.

### Data description

In our experiments, we use *Drosophila *gene expression pattern images retrieved from the FlyExpress database [8], which contains standardized versions of images obtained from the BDGP high-throughput study [1,2]. The images are standardized semi-manually, and all images are scaled to 128 × 320 pixels. The embryogenesis of *Drosophila *has been divided into six discrete stage ranges (stages 1–3, 4–6, 7–8, 9–10, 11–12, and 13–16) in the BDGP high-throughput study [1]. Since most of the CV terms are stage range specific, we annotate the images in each stage range separately. The *Drosophila *embryos are 3D objects, and the FlyExpress database contains 2D images that are taken from several different views (lateral, dorsal, ventral, and other intermediate views) of the 3D embryos. The size of the CV terms, the number of image groups, and the number of images with each view in each stage range are summarized in Table 1. We can observe that most of the images are taken from the lateral view. In stage range 13–16, the number of dorsal images is also comparable to that of the lateral images. We study the performance improvement obtained by using images with different views, and results show that incorporating images with dorsal views can improve performance consistently, especially in stage range 13–16 where the number of dorsal images is large. In contrast, the integration of ventral images results in marginal performance improvement at the price of an increased computational cost, since the number of ventral images is small. Hence, we only use the lateral and dorsal images in evaluating the relative performance of the compared methods.

### Evaluation of annotation performance

We apply the multi-label formulation proposed in [17] to annotate the gene expression pattern images. To demonstrate the effectiveness of this formulation in exploiting the correlation information among different labels, we also report the annotation performance achieved by the one-against-rest linear support vector machines (SVM) in which each linear SVM builds a decision boundary between image groups with and without one particular term. Note that in this method the labels are modeled separately, and hence no correlation information is captured. To compare the proposed method with existing approaches for this task, we report the annotation performance of a prior method [31], which used the pyramid match kernel (PMK) algorithm [32-34] to construct the kernel between two sets of feature vectors extracted from two sets of images. We report the performance of kernels constructed from the SIFT descriptor and that of the composite kernels combined from multiple kernels as in [31]. In the case of composite kernels, we apply the three kernel combination schemes (i.e., star, clique, and kCCA) and the best performance on each data set is reported. Note that the method proposed in [12] required that the training set contains embryos that are annotated individually, and it has been shown [31] that such requirement leads to low performance when applied to BDGP data in which the images are annotated in small groups. Hence, we do not report these results. In the following, the multi-label formulation proposed in [17] is denoted as ML_LS_, and the one-against-rest SVM is denoted as SVM. The pyramid match kernel approaches based on the SIFT and the composite features are denoted as PMK_SIFT _and PMK_comp_, respectively. All of the model parameters are tuned using 5-fold cross validation in the experiments.

From Table 1 we can see that the first stage range (1–3) is annotated with only two terms, and we do not report the results in this stage range. In other five stage ranges, we remove terms when they appeared in less than 5 training image groups in a stage range, which yielded data sets in which 60 or fewer terms need to be considered in every case. The two primary reasons for this decision are (1) terms which appeared in too few image groups are statistically too weak to be learned effectively, and (2) we used 5-fold cross-validation to tune the model parameters, and each term should appear in each fold at least once. Therefore, the maximum numbers of terms reported in Table 2, Table 3, Table 4, Table 5, and Table 6 represent the "all terms" test.

**Table 2:** Annotation performance in terms of AUC, macro F1, micro F1, sensitivity, and specificity for image groups in stage range 4–6.

Measure	# of terms	ML_LS_	SVM	PMK_SIFT_	PMK_comp_
AUC	10	80.85 ± 0.74	78.82 ± 0.78	79.15 ± 0.74	77.47 ± 0.80
	20	82.09 ± 0.52	80.33 ± 0.53	79.23 ± 0.56	77.46 ± 0.72
	30	79.30 ± 1.02	77.20 ± 1.01	76.21 ± 0.97	74.71 ± 0.84

macro F1	10	47.37 ± 1.55	46.59 ± 1.32	37.43 ± 2.03	43.08 ± 1.19
	20	39.38 ± 1.48	38.23 ± 1.16	25.25 ± 1.66	31.40 ± 1.37
	30	29.56 ± 1.34	28.75 ± 0.89	17.04 ± 0.96	22.13 ± 1.25

micro F1	10	50.75 ± 1.30	47.66 ± 1.35	45.91 ± 2.08	47.90 ± 1.00
	20	44.20 ± 1.22	41.31 ± 1.07	37.33 ± 1.17	40.88 ± 0.89
	30	41.88 ± 1.13	39.45 ± 0.85	34.27 ± 1.19	39.37 ± 0.99

Sensitivity	10	51.49 ± 2.49	60.42 ± 2.95	34.62 ± 2.42	52.04 ± 1.65
	20	39.84 ± 1.76	53.91 ± 1.95	21.28 ± 1.30	33.60 ± 1.38
	30	30.40 ± 1.93	40.27 ± 1.71	14.14 ± 0.83	24.79 ± 1.19

Specificity	10	86.56 ± 0.98	79.57 ± 1.86	92.56 ± 0.55	86.26 ± 0.62
	20	92.07 ± 0.62	85.81 ± 1.11	96.50 ± 0.29	93.33 ± 0.34
	30	94.53 ± 0.46	89.22 ± 0.89	97.73 ± 0.21	95.49 ± 0.28

"ML_LS_" denotes the performance obtained by applying the shared subspace multi-label formulation to the proposed bag-of-words representations derived from lateral and dorsal images. "SVM" denotes the performance of SVM applied on the bag-of-words representations using the one-against- rest scheme. "PMK" denotes the method based on pyramid match kernels, and the subscripts "SIFT" and "comp" denote kernels based on the SIFT descriptor and the composite kernels, respectively. Some terms appear in few image groups, and we eliminate them from the experiments. We randomly generate 30 different training/test partitions, and the average performance and standard deviation are reported.

**Table 3:** Annotation performance in terms of AUC, macro F1, micro F1, sensitivity, and specificity for image groups in stage range 7–8.

Measure	# of terms	ML_LS_	SVM	PMK_SIFT_	PMK_comp_
AUC	10	74.61 ± 1.03	73.65 ± 0.91	70.50 ± 1.37	72.35 ± 0.90
	20	72.23 ± 1.14	72.05 ± 1.30	68.36 ± 1.79	69.79 ± 1.18

macro F1	10	48.16 ± 1.43	48.03 ± 1.07	37.95 ± 1.06	46.35 ± 1.11
	20	30.54 ± 0.87	32.37 ± 1.04	19.87 ± 1.00	26.92 ± 1.03

micro F1	10	55.08 ± 1.31	53.36 ± 1.24	52.27 ± 1.09	52.65 ± 1.39
	20	52.27 ± 1.43	50.25 ± 1.32	48.38 ± 1.32	49.57 ± 1.34

Sensitivity	10	53.22 ± 2.94	63.77 ± 2.89	40.17 ± 1.45	54.21 ± 1.93
	20	32.32 ± 1.49	43.04 ± 1.77	20.22 ± 0.92	30.97 ± 1.38

Specificity	10	77.40 ± 1.91	71.23 ± 2.33	82.88 ± 1.01	82.21 ± 1.03
	20	89.25 ± 1.14	84.50 ± 1.17	92.34 ± 0.51	91.32 ± 0.62

See the caption of Table 2 for detailed explanations.

**Table 4:** Annotation performance in terms of AUC, macro F1, micro F1, sensitivity, and specificity for image groups in stage range 9–10.

Measure	# of terms	ML_LS_	SVM	PMK_SIFT_	PMK_comp_
AUC	10	77.16 ± 0.55	74.89 ± 0.68	72.08 ± 1.02	72.28 ± 0.72
	20	78.34 ± 0.85	76.38 ± 0.84	72.02 ± 1.56	72.75 ± 0.99

macro F1	10	53.43 ± 1.02	52.25 ± 0.98	39.46 ± 1.22	47.06 ± 1.16
	20	34.79 ± 1.12	35.62 ± 0.99	21.47 ± 0.75	28.21 ± 1.00

micro F1	10	59.96 ± 0.93	55.74 ± 1.02	53.43 ± 0.86	54.11 ± 0.95
	20	55.36 ± 0.95	51.70 ± 1.17	48.78 ± 0.93	49.80 ± 1.09

Sensitivity	10	55.36 ± 2.39	65.10 ± 2.29	41.25 ± 1.20	53.47 ± 1.81
	20	35.42 ± 1.12	45.73 ± 1.57	21.35 ± 0.71	33.49 ± 1.10

Specificity	10	77.98 ± 1.84	72.13 ± 2.28	82.30 ± 1.07	80.53 ± 1.25
	20	91.19 ± 0.51	85.45 ± 1.11	92.86 ± 0.35	91.61 ± 0.50

See the caption of Table 2 for detailed explanations.

**Table 5:** Annotation performance in terms of AUC, macro F1, micro F1, sensitivity, and specificity for image groups in stage range 11–12.

Measure	# of terms	ML_LS_	SVM	PMK_SIFT_	PMK_comp_
AUC	10	84.24 ± 0.54	83.05 ± 0.54	79.41 ± 0.58	78.68 ± 0.58
	20	84.23 ± 0.37	82.73 ± 0.38	77.62 ± 0.48	76.97 ± 0.67
	30	81.32 ± 0.44	79.18 ± 0.51	73.26 ± 0.42	72.90 ± 0.63
	40	75.68 ± 0.68	77.52 ± 0.63	72.96 ± 0.98	72.12 ± 0.64
	50	77.87 ± 0.81	76.19 ± 0.72	71.55 ± 1.14	70.73 ± 0.83

macro F1	10	61.07 ± 0.99	60.37 ± 0.88	47.22 ± 1.44	55.19 ± 0.62
	20	48.06 ± 0.80	46.03 ± 0.83	26.36 ± 0.85	36.11 ± 0.88
	30	34.92 ± 0.99	35.32 ± 0.75	18.00 ± 0.64	24.85 ± 0.63
	40	26.14 ± 0.65	27.85 ± 0.79	13.31 ± 0.47	19.01 ± 0.63
	50	22.77 ± 1.00	23.46 ± 0.60	10.79 ± 0.41	15.04 ± 0.46

micro F1	10	66.77 ± 0.72	65.67 ± 0.60	60.41 ± 0.93	62.55 ± 0.68
	20	59.83 ± 0.65	54.59 ± 0.87	50.49 ± 0.71	53.61 ± 0.59
	30	54.99 ± 0.79	48.87 ± 0.85	47.01 ± 0.73	49.61 ± 0.64
	40	49.49 ± 0.71	46.40 ± 0.91	45.38 ± 0.61	48.10 ± 0.75
	50	52.15 ± 0.67	47.18 ± 0.84	44.95 ± 0.56	47.41 ± 0.61

Sensitivity	10	66.99 ± 1.17	70.18 ± 1.78	46.92 ± 1.44	64.43 ± 0.96
	20	48.35 ± 1.31	55.43 ± 1.56	24.81 ± 0.69	43.39 ± 1.22
	30	34.80 ± 1.33	48.99 ± 1.87	16.64 ± 0.45	31.00 ± 0.97
	40	26.96 ± 1.20	37.42 ± 1.33	12.35 ± 0.32	23.49 ± 0.96
	50	22.78 ± 1.11	30.36 ± 1.24	10.01 ± 0.30	18.68 ± 0.69

Specificity	10	80.84 ± 1.19	77.58 ± 1.29	84.11 ± 0.86	80.93 ± 0.78
	20	92.00 ± 0.52	87.85 ± 0.78	93.93 ± 0.30	92.76 ± 0.29
	30	94.33 ± 0.40	88.67 ± 0.92	96.38 ± 0.19	95.52 ± 0.21
	40	94.80 ± 0.43	92.10 ± 0.56	97.30 ± 0.14	96.65 ± 0.15
	50	96.29 ± 0.25	93.80 ± 0.45	97.85 ± 0.10	97.28 ± 0.12

See the caption of Table 2 for detailed explanations.

**Table 6:** Annotation performance in terms of AUC, macro F1, micro F1, sensitivity, and specificity for image groups in stage range 13–16.

Measure	# of terms	ML_LS_	SVM	PMK_SIFT_	PMK_comp_
AUC	10	87.72 ± 0.37	86.66 ± 0.35	82.51 ± 0.43	82.53 ± 0.62
	20	84.61 ± 0.35	83.25 ± 0.35	76.62 ± 0.41	77.09 ± 0.55
	30	82.92 ± 0.45	81.13 ± 0.46	73.13 ± 0.61	74.05 ± 0.68
	40	77.25 ± 0.40	79.56 ± 0.32	70.37 ± 0.43	71.48 ± 0.45
	50	79.38 ± 0.50	78.17 ± 0.38	68.52 ± 0.50	69.15 ± 0.73
	60	78.11 ± 0.55	77.18 ± 0.46	67.41 ± 0.68	68.24 ± 0.48

macro F1	10	64.91 ± 0.82	62.97 ± 0.68	54.51 ± 0.88	58.42 ± 0.94
	20	49.90 ± 0.75	50.45 ± 0.62	31.61 ± 0.99	41.02 ± 0.72
	30	42.57 ± 0.78	41.92 ± 0.76	21.28 ± 0.62	31.04 ± 0.82
	40	32.26 ± 0.57	35.26 ± 0.63	15.75 ± 0.39	23.78 ± 0.48
	50	29.13 ± 0.66	29.74 ± 0.45	12.65 ± 0.40	19.22 ± 0.53
	60	25.18 ± 0.68	25.49 ± 0.55	10.56 ± 0.31	16.13 ± 0.48

micro F1	10	68.27 ± 0.52	66.67 ± 0.45	60.98 ± 0.69	62.07 ± 0.90
	20	57.62 ± 0.51	55.66 ± 0.65	48.75 ± 0.61	50.56 ± 0.59
	30	53.02 ± 0.68	48.11 ± 0.90	42.88 ± 0.66	45.48 ± 0.82
	40	46.92 ± 0.55	44.26 ± 0.92	39.77 ± 0.43	42.55 ± 0.67
	50	48.76 ± 0.53	43.53 ± 0.77	38.55 ± 0.69	41.07 ± 0.89
	60	48.02 ± 0.61	42.84 ± 0.76	37.77 ± 0.57	40.65 ± 0.49

Sensitivity	10	70.14 ± 1.39	70.21 ± 1.53	51.20 ± 0.97	67.21 ± 1.30
	20	52.99 ± 1.57	61.45 ± 1.22	28.55 ± 0.98	46.84 ± 1.05
	30	44.46 ± 1.26	55.56 ± 1.17	19.02 ± 0.60	35.43 ± 0.75
	40	34.04 ± 1.18	48.94 ± 1.78	14.07 ± 0.38	27.93 ± 0.63
	50	29.51 ± 0.94	40.45 ± 1.20	11.25 ± 0.39	24.04 ± 0.63
	60	25.25 ± 0.92	34.53 ± 1.04	9.36 ± 0.30	21.28 ± 0.51

Specificity	10	85.89 ± 0.61	83.65 ± 0.87	89.31 ± 0.50	84.17 ± 0.86
	20	90.32 ± 0.56	86.31 ± 0.67	94.69 ± 0.22	91.16 ± 0.48
	30	92.93 ± 0.31	87.65 ± 0.67	96.78 ± 0.15	94.38 ± 0.37
	40	93.88 ± 0.36	89.38 ± 0.78	97.64 ± 0.12	95.84 ± 0.21
	50	95.62 ± 0.23	91.50 ± 0.52	98.18 ± 0.10	96.59 ± 0.29
	60	96.38 ± 0.19	92.74 ± 0.38	98.50 ± 0.08	97.23 ± 0.12

See the caption of Table 2 for detailed explanations.

The experiments are geared toward examining the change in the accuracy of our annotation method, as we used an increasingly larger set of vocabulary terms. In our experiment, we begin with the 10 terms that appear in the largest number of image groups. Then we add additional terms in the order of their frequencies. By virtue of this design, experiments with 10 terms should show higher performance than those with 50 terms, because 10 most frequent terms will appear more often in image groups in the training data sets as compared to the case of 50 terms (for example). The extracted data set is partitioned into training and test sets using the ratio 1:1 for each term, and the training data are used to construct the classification model.

**Figure 5:**
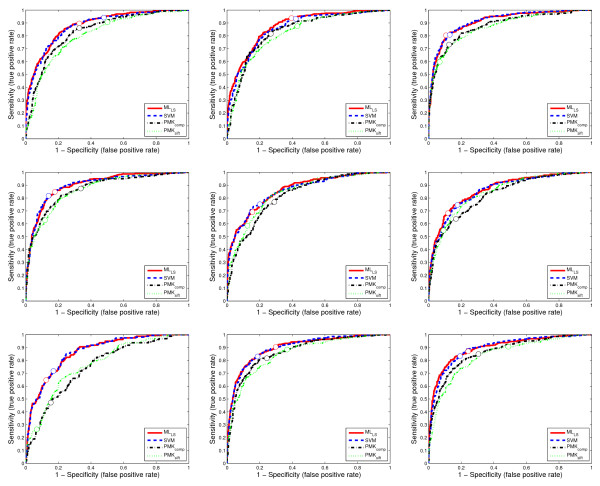
**The ROC curves for 9 randomly selected terms on a data set from stage range 11–12**. Each figure corresponds to the ROC curves for a term. The circles on the curves show the corresponding decision points, which are tuned on the training set based on F1 score.

**Figure 6:**
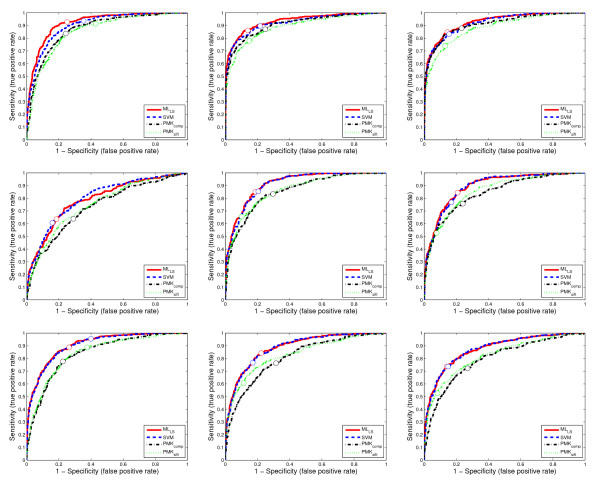
**The ROC curves for 9 randomly selected terms on a data set from stage range 13–16**. Each figure corresponds to the ROC curves for a term. The circles on the curves show the corresponding decision points, which are tuned on the training set based on F1 score.

The agreement between the predicted annotations and the expert data provided by human curators is measured using the area under the receiver operating characteristic (ROC) curve, called AUC [35], F1 measure [36], sensitivity and specificity. For AUC, the value for each term is computed and the averaged performance across multiple terms is reported. For F1 measure, there are two ways, called *macro*-averaged F1 and *micro*-averaged F1, respectively, to average the performance across multiple terms and we report both results. For each data set, the training and test data sets are randomly generated 30 times, and the averaged performance and standard deviations are reported in Table 2, Table 3, Table 4, Table 5, and Table 6. To compare the performance of all methods across different values of sensitivity and specificity, we show the ROC curves of 9 randomly selected terms on two data sets from stage ranges 11–12 and 13–16 in Figures 5 and 6.

We can observe from Table 2, Table 3, Table 4, Table 5, and Table 6 and Figures 5 and 6 that approaches based on the bag-of-words representation (ML_LS _and SVM) consistently outperform the PMK-based approaches (PMK_SIFT _and PMK_comp_). Note that since both the shared-subspace formulation and SVM are based on the bag-of-words representation, the benefit of this representation should be elucidated by comparing the performance of both the shared-subspace formulation and SVM to the two approaches based on PMK. In particular, ML_LS _outperforms PMK_SIFT _and PMK_comp _on all of the 18 data sets in terms of all three performance measures (AUC, macro F1, and micro F1). In all cases, the performance improvements tend to be larger for the two F1 measures than AUC. It can also be observed from Figures 5 and 6 that the ROC curves for SVM and the shared-subspace formulation are always above those based on the pyramid match algorithm. This indicates that both SVM and the shared-subspace formulation outperform previous methods across all classification thresholds. A similar trend has been observed from other data sets, but their detailed results are omitted due to space constraint. This shows that the bag-of-words scheme is more effective in representing the image groups than the PMK-based approach. Moreover, we can observe that ML_LS _outperforms SVM on most of the data sets for all three measures. This demonstrates that the shared-subspace multi-label formulation can improve performance by capturing the correlation information among different labels. For the PMK-based approaches, PMK_comp _outperforms PMK_SIFT _on all of the data sets. This is consistent with the prior results obtained in [31] that the integration of multiple kernel matrices derived from different features improves performance.

### Performance of individual terms

**Figure 7:**
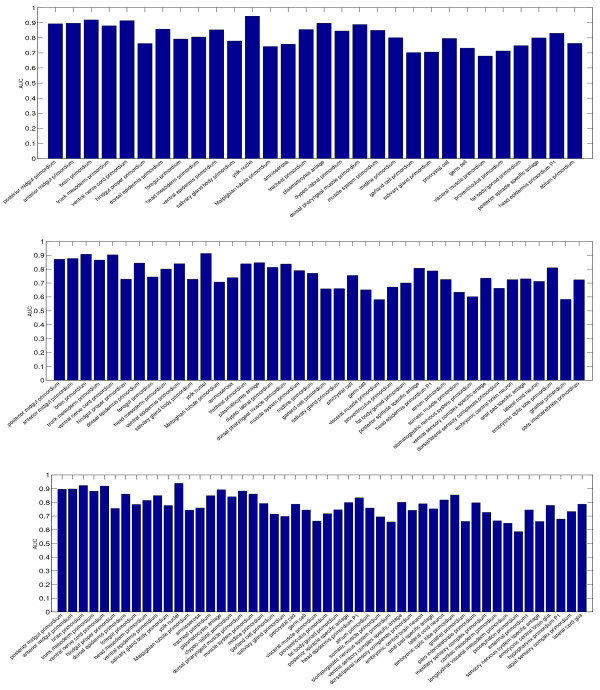
**The AUC of individual terms on three data sets from stage range 11–12**. The three figures, from top to down, show the performance on data sets with 30, 40, and 50 terms, respectively.

**Figure 8:**
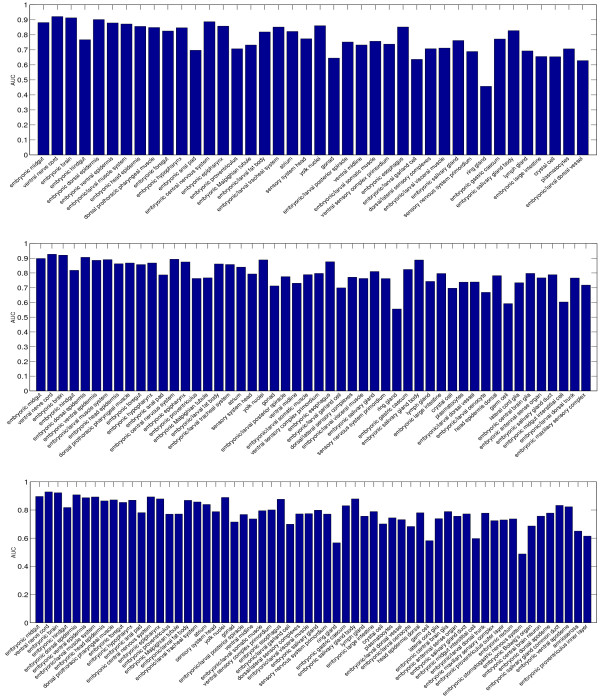
**The AUC of individual terms on three data sets from stage range 13–16**. The three figures, from top to down, show the performance on data sets with 40, 50, and 60 terms, respectively.

To evaluate the relative performance of the individual terms used, we report the AUC values achieved by the proposed formulation on 6 data sets in Figures 7 and 8. One major outcome of our analysis was that some terms were consistently assigned to wrong image groups. For example, the terms "hindgut proper primordium", "Malpighian tubule primordium", "garland cell primordium", "salivary gland primordium", and "visceral muscle primordium" in stage range 11–12 achieve low AUC on all three data sets. Similarly, the terms "ring gland", "embryonic anal pad", "embryonic proventriculus", "gonad"", and "embryonic/larval garland cell" achieve low AUC on all three data sets in stage range 13–16. For most of these terms, the low performance is caused by the fact that they only appear in very few image groups. Such low frequencies result in weak learning due to statistical reasons. Therefore, the number of images available for training our method will need to be increased to improve performance.

### Integration of images with multiple views

**Figure 9:**
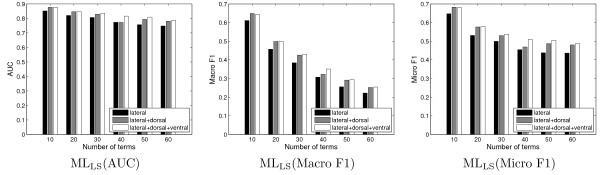
**Comparison of annotation performance achieved by ML_LS _when images from different views (lateral, lateral+dorsal, and lateral+dorsal+ventral) are used on 6 data sets from the stage range 13–16**. In each figure, the x-axis denotes the data sets with different numbers of terms. For each data set, 30 random partitions of the training and test sets are generated and the averaged performance is reported.

To evaluate the effect of integrating images with multiple views, we report the annotation performance in the cases of using only lateral images, lateral and dorsal images, and lateral, dorsal, and ventral images. In particular, we extract six data sets from the stage range 13–16 with the number of terms ranged from 10 to 60 with a step size of 10. The average performance in terms of AUC, macro F1, and micro F1 achieved by ML_LS _over 30 random trials is shown in Figure 9. We observe that performance can be improved significantly by incorporating the dorsal view images. In contrast, the incorporation of ventral images results in slight performance improvement. In other stage ranges, the integration of images with multiple views can either improve or keep comparable performance. This may be due to the fact that the dorsal view images are mostly informative for annotating embryos in stage range 13–16, as large morphological movements happen on dorsal side in this stage range. Similar trends have been observed when the SVM classifier is applied.

### Effect of codebook size

**Figure 10:**
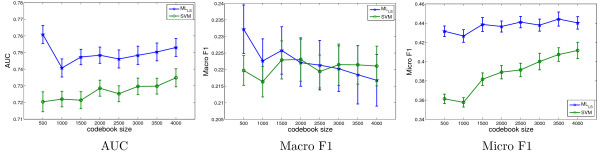
**The change of performance when the codebook size increases gradually from 500 to 4000 with a step size of 500 on a data set in stage range 13–16 with 60 terms**. In each case, the average performance and standard deviation over 30 random partitions of the training and test sets are shown. A similar trend has been observed in other stage ranges.

The size of the codebook is a tunable parameter, and we evaluate its effect on annotation performance using a subset of lateral images from stage range 13–16 with 60 terms in this experiment. In particular, the size of the codebook for this data set increases from 500 to 4000 gradually with a step size of 500, and the performance of ML_LS _and SVM is plotted in Figure 10. In most cases the performance can be improved with a larger codebook size, but it can also decrease in certain cases such as the performance of ML_LS _when measured by macro F1. In general, the performance does not change significantly with codebook size. Hence, we set the codebook size to 2000 for lateral images in previous experiments to maximize performance and minimize computational cost. An interesting observation from Figure 10 is that the performance differences between ML_LS _and SVM tend to be larger for a small codebook size. This may reflect the fact that small codebook sizes cannot capture the complex patterns in image groups. This representation insufficiency can be compensated effectively by sharing information between image groups using the shared-subspace multi-label formulation. For a large codebook size, the performance of ML_LS _and SVM tend to be close.

## Conclusion

In this article we present a computational method for automated annotation of *Drosophila *gene expression pattern images. This method represents image groups using the bag-of-words approach and annotates the groups using a shared-subspace multi-label formulation. The proposed method annotates images in groups, and hence retains the image group membership information as in the original BDGP study. Moreover, multiple sources of information conveyed by images with different views can be integrated naturally in the proposed method. Results on images from the FlyExpress database demonstrate the effectiveness of the proposed method.

In constructing the bag-of-words representation in this article, we only use the SIFT features. Prior results on other image-related applications show that integration of multiple feature types may improve performance [37]. We plan to extend the proposed method for integrating multiple feature types in the future. In addition, the bag-of-words representation is obtained by the hard assignment approach in which a local feature vector is only assigned to the closest visual word. Recent study [38] shows that the soft assignment approach that assigns each feature vector to multiple visual words based on their distances usually results in improved performance. We will explore this in the future.

## Authors' contributions

All authors analyzed the results and wrote the paper. SJ designed the methodology, implemented the programs, and drafted the manuscript. SK and JY supervised the project and guided the implementation. All authors have read and approved the final manuscript.
